# Incidence Patterns of Primary Bone Cancer in Taiwan (2003–2010): A Population-Based Study

**DOI:** 10.1245/s10434-014-3697-3

**Published:** 2014-04-11

**Authors:** Giun-Yi Hung, Jiun-Lin Horng, Hsiu-Ju Yen, Chueh-Chuan Yen, Wei-Ming Chen, Paul Chih-Hsueh Chen, Hung-Ta Hondar Wu, Hong-Jen Chiou

**Affiliations:** 1 Division of Pediatric Hematology and Oncology, Department of Pediatrics, Taipei Veterans General Hospital, Taipei, Taiwan, Republic of China; 2 Therapeutical and Research Center of Musculoskeletal Tumor, Taipei Veterans General Hospital, Taipei, Taiwan; 3 School of Medicine, National Yang-Ming University, Taipei, Taiwan; 4 Department of Anatomy, Taipei Medical University, Taipei, Taiwan; 5 Division of Hematology and Oncology, Department of Medicine, Taipei Veterans General Hospital, Taipei, Taiwan; 6 Departments of Orthopedics, Taipei Veterans General Hospital, Taipei, Taiwan; 7 Department of Pathology, Taipei Veterans General Hospital, Taipei, Taiwan; 8 Department of Radiology, Taipei Veterans General Hospital, Taipei, Taiwan; 9 National Defense Medical Center, Taipei, Taiwan

## Abstract

**Background:**

Primary bone cancer (BC) incidence by age has not been surveyed in Asia.

**Methods:**

The incidence patterns of nine subtypes of primary BCs registered between 2003 and 2010 were analyzed from Taiwan cancer registry data. More specific analyses were conducted within age groups (Group I: 0–24 years; Group II: 25–59 years; and Group III: 60–85+ years).

**Results:**

A total of 1,238 newly diagnosed subjects were registered with an age-standardized incidence rate (ASR) of 6.70 per million person-years. Overall, osteosarcoma (OS: 45 %) was the most common, followed by chondrosarcoma (CS: 18 %), and Ewing sarcoma (ES: 8 %). The percentages of cases and ASRs for age groups I, II, and III were 36.3, 43.0, and 20.7 %, and 7.00, 5.48, and 10.28 per million, respectively. Significant male predilections were observed for all BCs combined, and the CS, chordoma, and malignant ameloblastoma subtypes. Our findings demonstrated an upward trend of 4.8 % per year over the study period, and was more significant for females (6.7 %). A significant increase in trend existed in the incidence of BC among females in Group II, and the incidence of OS and ES among females in Group I.

**Conclusions:**

This population-based study has allowed us to confidently define the incidence rates among three age groups of Taiwanese. Despite overall low rates, the upward trend in BC incidence among females may invoke a concern. The results suggest areas for further study into the underlying causes for these cancer trends.

Primary bone cancer (BC) is rare, comprising less than 1 % of all cancers; however, it is the seventh most common type of cancer among children and adolescents, representing 5 % of cancers in those aged 0–19 years.^1^ Previous studies from Western countries have indicated significant differences in the incidence of BC subtypes among different ethnic groups.^2^–^5^ Because the incidence of BC is clearly age-dependent, the incidence of BC analyzed by age groups would be more meaningful.^2^,^5^ A study on worldwide osteosarcoma (OS) incidence patterns revealed that incidence rates were similar in the younger age groups, while the greatest variation was observed in the elderly. To our knowledge, reliable data on the incidence in elderly patients of some Asian countries is lacking.^5^ A previous report on the cancer trends in Taiwan showed the incidence of BC was based on the population of all ages combined, but without data by age groups.^6^

Most studies investigating pediatric groups enrolled patients aged 0–18 years; however, primary BCs were not only common in adolescents (15–19 years) but also in young adults (20–24 years), and usually they share common treatment strategies in both age groups. Surveillance of cancer incidence in this neglected age group of young adults would raise awareness about primary BC. The current study was based on the population-based data from the Taiwan cancer registry (TCR) and aimed to broaden the availability and comparability of data and characterization of BC incidence in all age groups. Three age groups (0–24, 25–59, and 60–85+ years) were categorized to focus on the two incidence peaks in children/adolescents and the elderly, as well as the incidence plateau among subjects aged 25–59 years as described previously.^2^,^5^ Moreover, because up to 98 % of the population (23 million) of Taiwan are Han Chinese,^7^ the results from this study will provide further insights into the comparisons of other Chinese ethnic groups with populations from Western countries.

## Materials and Methods

### Data Collection

Incidence data were obtained from the TCR, which is organized and funded by the Health Promotion Administration, Ministry of Health and Welfare, Taiwan. The TCR is population-based and began data collection in 1979. Taiwan’s National Health Insurance program was first launched in 1995, which is a mandatory universal health insurance program with a coverage rate of up to 99 %. Furthermore, in accordance with the enactment of the Cancer Control Act in 2003, hospitals with a capacity of more than 50 beds were mandated to submit cancer data to the central cancer registry, which enhanced the completeness of case ascertainment and improved the quality of cancer data collection.^6^,^8^ In terms of data quality of the TCR according to the indicators defined by the International Agency for Research on Cancer of the World Health Organization, the percentage of ‘death certificate only’ cases fell from 2.66 % in 2003 to 0.85 % in 2010.^8^ The percentage of microscopically verified cases (MV%) is another indicator presenting data validity; despite varying by the types of cancer, the percentage was 91.1 % in 2010 for all cancers combined. The above indicators reveal the high quality of the registry, with remarkable improvement over time.

Primary BCs were classified according to the International classification of diseases for oncology, third edition (ICD-O-3: C40–C41).^9^ Subgroups of primary BCs were defined by the TCR as ‘osteosarcoma’, ‘chondrosarcoma (CS)’, ‘malignant giant cell tumor (GCT)’, ‘Ewing sarcoma (ES)’, ‘chordoma’, ‘malignant ameloblastoma’, ‘other specified sarcoma (including fibrosarcoma, synovial sarcoma, hemangiosarcoma, neurofibrosarcoma, etc.)’, ‘sarcoma, not otherwise specified (NOS: including undifferentiated sarcoma and malignant tumor, fusiform cell type, etc.)’, and ‘other malignancy (including carcinomas and other non-sarcoma neoplasms)’. Lymphoma and multiple myeloma were not included in the current study.

### Analyses

Age-standardized incidence rates (ASRs) were expressed per million person-years by gender, and presented according to the nine subgroups, as defined above. Rates, cumulative risk, standard errors, and 95 % confidence intervals (CIs) were calculated by the methods published previously.^10^ Age-specific rates were stratified into the following eighteen 5-year age groups: 0–4, 5–9, 10–14, 15–19, 20–24, …, and 85+. ASRs were computed by the direct method of standardization according to the 2000 world standard population for 5-year age groups.^10^ More specific comparison of the ASRs among the three age groups (Group I: 0–24 years; Group II: 25–59 years; and Group III: 60–85+ years) were performed. Male-to-female standardized rate ratios (M/F SRRs) and 95 % CIs were calculated by histologic subtypes for the three age groups.^10^ The rates were considered to be significantly different at the 5 % level when the estimated 95 % CI did not contain 1. The results of quality indicators, including the MV% by subtypes, were also analyzed. Trend analyses were calculated by the Joinpoint regression model, and permutation tests were used to determine the significance level (Joinpoint Regression Program, Version 4.0.4).^11^ Results were expressed as the average annual percent change (AAPC). The AAPC was considered significant if the 95 % CI did not include zero.

## Results

A total of 1,238 subjects were diagnosed with primary BCs between 2003 and 2010, giving a crude rate of 6.78 and ASR of 6.70 per million person-years, respectively (Table 1). Primary BCs comprised 0.2 % of all cancers. The median age of patients at diagnosis was 39 years. The cumulative risk of developing primary BC from birth to age 74 years was 0.05 %. OS was the most common BC subtype, accounting for 45 %, followed by CS (18 %) and ES (8 %). The three subtypes comprised approximately 70 % of primary BCs. Overall, the MV% for primary BCs was 96.0 %. The MV% varied from 97.5 to 100 % for all BC subtypes with the exception of ‘other malignancy’, in which the MV% only accounted for 29.8 %.

**Table 1:** Incidence of primary bone cancer by age group, gender, and histologic subtype, Taiwan (2003–2010)

Histologic subtype	Gender	Age group (years)											
		I: 0–24			II: 25–59			III: ≥60			All ages		
		No.	ASR^a^	95 % CI	No.	ASR^a^	95 % CI	No.	ASR^a^	95 % CI	No.	ASR^a^	95 % CI
Osteosarcoma	All persons	300	4.61	4.09–5.14	160	1.66	1.41–1.92	59	2.41	1.79–3.03	519	3.01	2.75–3.28
	Males	173	5.17	4.39–5.94	74	1.52	1.17–1.86	29	2.27	1.43–3.10	276	3.17	2.79–3.55
	Females	127	4.17	3.44–4.89	86	1.75	1.38–2.13	30	2.39	1.53–3.25	243	2.86	2.49–3.23
Chondrosarcoma	All persons	30	0.45	0.28–0.61	155	1.60	1.35–1.85	59	2.37	1.76–2.99	244	1.20	1.05–1.35
	Males	21	0.63	0.36–0.90	85	1.72	1.35–2.08	31	2.43	1.56–3.30	137	1.34	1.11–1.56
	Females	9	0.27	0.09–0.44	70	1.42	1.08–1.75	28	2.19	1.37–3.01	107	1.02	0.82–1.21
Ewing sarcoma	All persons	54	0.89	0.65–1.13	25	0.26	0.16–0.36	4	0.16	0.00–0.32	83	0.52	0.40–0.63
	Males	34	1.09	0.72–1.46	13	0.26	0.12–0.40	1	0.06	−0.06 to 0.19	48	0.59	0.42–0.76
	Females	20	0.72	0.40–1.04	12	0.25	0.11–0.39	3	0.24	−0.03 to 0.51	35	0.45	0.29–0.60
Malignant giant cell tumor	All persons	6	0.09	0.02–0.16	24	0.25	0.15–0.36	10	0.44	0.17–0.72	40	0.20	0.14–0.27
	Males	2	0.06	−0.02 to 0.13	12	0.25	0.11–0.40	6	0.54	0.11–0.97	20	0.20	0.11–0.29
	Females	4	0.12	0.00–0.24	12	0.25	0.11–0.39	4	0.33	0.00–0.66	20	0.21	0.11–0.30
Chordoma	All persons	6	0.09	0.02–0.16	48	0.48	0.34–0.62	29	1.17	0.74–1.60	83	0.40	0.31–0.48
	Males	4	0.12	0.00–0.24	32	0.64	0.41–0.86	20	1.54	0.85–2.23	56	0.52	0.38–0.66
	Females	2	0.06	−0.02 to 0.15	16	0.30	0.15–0.45	9	0.73	0.25–1.21	27	0.25	0.15–0.35
Malignant ameloblastoma	All persons	3	0.05	−0.01 to 0.10	16	0.16	0.08–0.25	10	0.41	0.15–0.67	29	0.14	0.09–0.20
	Males	2	0.06	−0.02 to 0.15	12	0.24	0.10–0.37	8	0.66	0.20–1.12	22	0.21	0.12–0.30
	Females	1	0.03	−0.03 to 0.09	4	0.09	0.00–0.17	2	0.16	−0.06 to 0.37	7	0.07	0.02–0.12
Other specified sarcoma	All persons	42	0.70	0.48–0.91	70	0.71	0.54–0.88	45	1.79	1.26–2.32	157	0.83	0.70–0.97
	Males	26	0.83	0.50–1.15	39	0.76	0.52–1.00	18	1.42	0.76–2.09	83	0.87	0.68–1.06
	Females	16	0.59	0.30–0.89	31	0.63	0.41–0.85	27	2.06	1.28–2.84	74	0.78	0.60–0.97
Sarcoma, NOS	All persons	4	0.07	0.00–0.13	17	0.17	0.09–0.25	15	0.57	0.28–0.87	36	0.17	0.12–0.23
	Males	2	0.07	−0.03 to 0.17	11	0.22	0.09–0.34	5	0.37	0.04–0.70	18	0.17	0.09–0.25
	Females	2	0.06	−0.02 to 0.15	6	0.12	0.02–0.21	10	0.75	0.28–1.22	18	0.17	0.09–0.25
Other malignancy	All persons	4	0.06	0.00–0.13	18	0.18	0.10–0.27	25	0.95	0.58–1.33	47	0.22	0.16–0.29
	Males	3	0.10	−0.02 to 0.22	11	0.23	0.09–0.36	13	0.91	0.41–1.41	27	0.25	0.16–0.35
	Females	1	0.03	−0.03 to 0.08	7	0.13	0.03–0.23	12	0.89	0.38–1.40	20	0.18	0.10–0.26
All types	All persons	449	7.00	6.35–7.65	533	5.48	5.02–5.95	256	10.28	9.01–11.55	1,238	6.70	6.32–7.08
	Males	267	8.12	7.13–9.10	289	5.83	5.15–6.50	131	10.20	8.42–11.97	687	7.33	6.77–7.89
	Females	182	6.05	5.17–6.94	244	4.94	4.31–5.56	125	9.74	8.02–11.46	551	5.99	5.47–6.50

*ASRs* age-standardized incidence rates, *CI* confidence interval, *NOS* not otherwise specified

^a^ASRs were per million person-years and were age-standardized to the 2000 world standard population

### Age-Specific Incidence Rates by Histologic Subtype

**Fig. 1:**
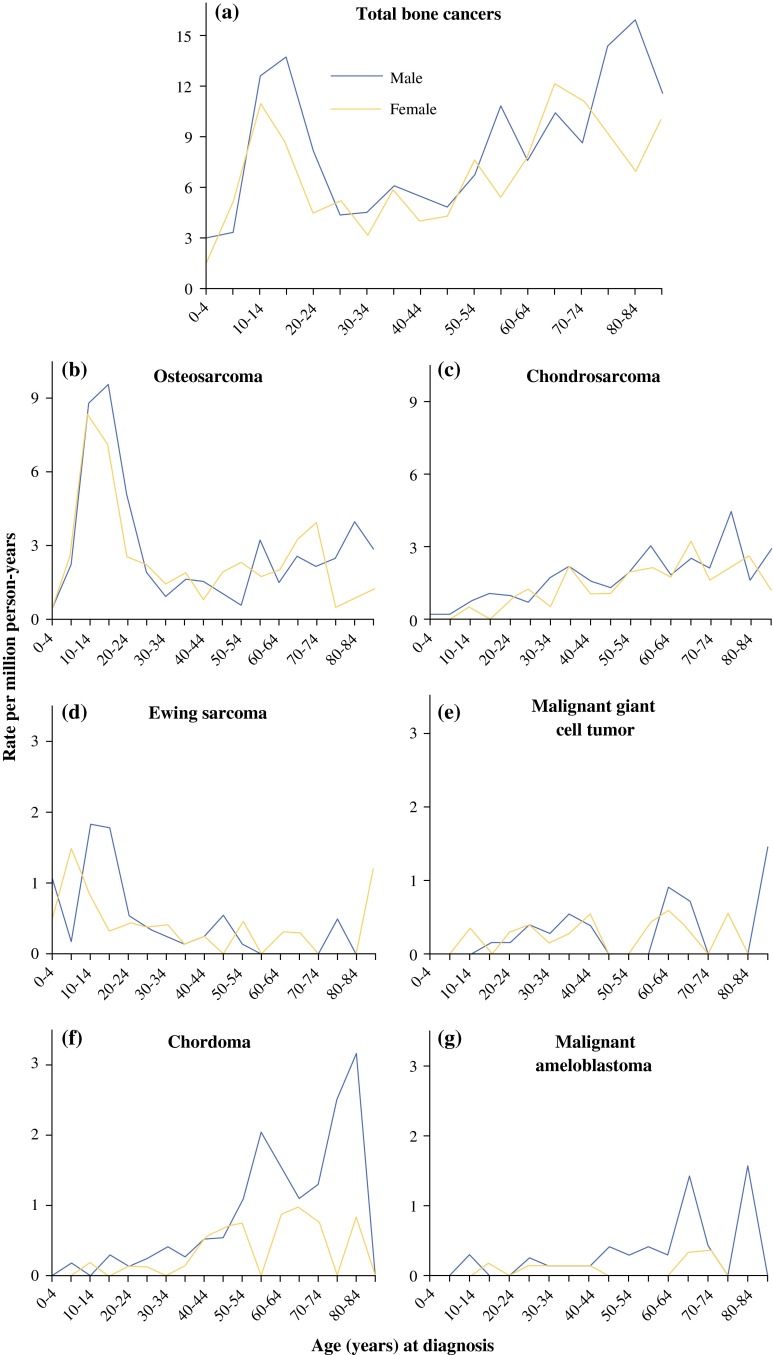
Age-specific incidence rates by histologic subtypes and gender for primary bone cancers in Taiwan (2003–2010)

The incidence of primary BCs revealed a bimodal distribution, with a primary peak during the second decade of life and a secondary peak amongst the elderly (Fig. 1a). The incidence patterns of OS and ES were similar and were more common for children and adolescents. OS peaked at 10–19 years of age for both genders, while ES peaked earlier, especially for females (5–9 years of age; Fig. 1b, d). The incidence of CS steadily increased with age (Fig. 1c). Malignant GCT was more common in middle-aged and elderly patients (Fig. 1e). Chordoma and malignant ameloblastoma mainly affected elderly individuals (Fig. 1f, g).

### Relative Rate of Histologic Subtypes in Age Groups I, II, and III

Patients with primary BCs accounted for 36.3, 43.0, and 20.7 % for age groups I, II, and III, respectively (Table 1). There were 449 patients with an ASR of 7.00 per million in Group I (Table 1). OS was the most common subtype, comprising two-thirds of the patients, and ES was the second most common subtype (13 %), followed by CS (6 %). Other specified sarcomas accounted for 10 % of the primary BCs. All other subtypes were rare.

Group II comprised of 533 patients, with an ASR of 5.48 per million. Together, OS (30 %) and CS (29 %) accounted for approximately 60 % of primary BCs and were the most common subtypes. Chordoma was the third most common subtype (9 %). ES, malignant GCT, and malignant ameloblastoma were rare.

Group III comprised of 256 patients, with an ASR of 10.28 per million. OS and CS were the most common primary BCs and the rates were similar (24–23 %, respectively). As with Group II, chordoma was also the third most common subtype and accounted for 11 % of primary BCs.

### Male-to-Female Standardized Rate Ratios by Histologic Subtype in Age Groups I, II, and III

For all age groups combined, males were more likely to be diagnosed with primary BCs than females (M/F SRR 1.22; Table 2). There was a significant male predilection in some subtypes, including CS, chordoma, and malignant ameloblastoma, with M/F SRRs ranging from 1.31 to 2.98. Malignant GCT was the only subtype in which the M/F SRR was <1; however, this result was not statistically significant.

**Table 2:** Male-to-female SRR by histologic subtype among three age groups for primary bone cancer, Taiwan (2003–2010)

Histologic subtype	Age group (years)							
	I: 0–24		II: 25–59		III: ≥60		All ages	
	SRR	95 % CI	SRR	95 % CI	SRR	95 % CI	SRR	95 % CI
Osteosarcoma	1.24	0.99–1.56	0.86	0.63–1.18	0.95	0.57–1.59	1.11	0.93–1.32
Chondrosarcoma	2.33*	1.09–5.01	1.21	0.88–1.66	1.11	0.66–1.86	1.31*	1.02–1.69
Ewing sarcoma	1.51	0.87–2.62	1.04	0.47–2.29	0.27	0.03–2.56	1.32	0.84–2.06
Malignant giant cell tumor	0.46	0.09–2.48	1.01	0.45–2.24	1.63	0.46–5.81	0.99	0.53–1.84
Chordoma	1.98	0.37–10.49	2.11*	1.16–3.81	2.11	0.97–4.56	2.09*	1.33–3.30
Malignant ameloblastoma	1.90	0.18–19.75	2.77	0.95–8.05	4.21	0.97–18.16	2.98*	1.32–6.69
Other specified sarcoma	1.39	0.75–2.60	1.22	0.76–1.95	0.69	0.38–1.26	1.11	0.80–1.54
Sarcoma, NOS	1.11	0.15–8.18	1.82	0.68–4.87	0.49	0.17–1.42	1.01	0.52–1.97
Other malignancy	3.59	0.37–34.85	1.75	0.68–4.53	1.02	0.46–2.24	1.43	0.79–2.59
All types	1.34*	1.11–1.62	1.18	1.00–1.40	1.05	0.82–1.34	1.22*	1.09–1.37

*SRR* standardized rate ratio, *CI* confidence interval, *NOS* not otherwise specified

* Indicates statistical significance at the 0.05 level

A significant male predominance was apparent in Group I when all patients were combined, with a M/F SRR of 1.34. In the subtypes of BCs, only CS exhibited a significant male predominance (M/F SRR 2.33).

While incidence rates for all patients of subtypes in Groups II and III combined, no statistical difference was detected between genders, except chordomas in Group II with male predominance (M/F SRR 2.11).

### Temporal Trends

**Fig. 2:**
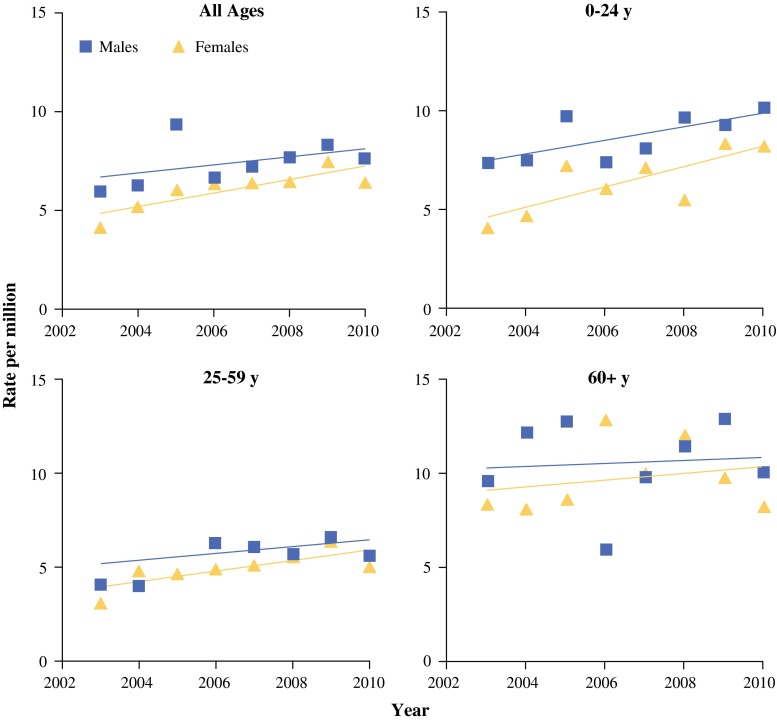
Temporal trends in incidence rates of primary bone cancer by age group in Taiwan (2003–2010)

Figure 2 illustrates the temporal trends in the incidence rates by age groups during the study period. Table 3 demonstrates the AAPC in incidence rates by age group, histologic subtype, and gender during 2006–2010. For all age groups combined, the AAPC of all BCs was 4.8 % with statistical significance, and was more significant for females (AAPC 6.7 %). Furthermore, the incidence rates rose significantly in subtypes of OS, ES, and sarcoma NOS (AAPC 5.5–43.0 %).

**Table 3:** AAPC in incidence rates by histologic subtype and gender, primary bone cancer, Taiwan (2006–2010)

Histologic subtype	Gender	Age group (years)							
		I: 0–24		II: 25–59		III: ≥60		All ages	
		AAPC^a^	95 % CI	AAPC^a^	95 % CI	AAPC^a^	95 % CI	AAPC^a^	95 % CI
Osteosarcoma	All persons	3.3	−0.5 to 7.2	4.9	−1.4 to 11.6	7.3	−6.9 to 23.8	5.5*	4.2–6.9
	Males	4.6*	0.9–8.4	−4.7	−11.2 to 2.3	3.1	−11.1 to 19.5	0.9	−2.0 to 4.0
	Females	14.0*	7.6–20.7	7.5	−2.9 to 19.0	–		9.4*	4.7–14.2
Chondrosarcoma	All persons	−5.5	−21.3 to 13.6	8.3	−2.1 to 19.7	8.2*	0.9–16.1	6.0	−2.2 to 14.9
	Males	–		3.0	−7.7 to 14.9	8.7	−6.2 to 26.0	5.7	−2.4 to 14.6
	Females	–		6.8	−6.4 to 21.9	−2.9	−17.8 to 14.7	4.4	−6.1 to 16.1
Ewing sarcoma	All persons	4.5	−12.8 to 25.2	–		–		43.0*	40.1–45.9
	Males	–		–		–		–	
	Females	14.6*	0–31.4	–		–		14.4	−0.3 to 31.3
Malignant giant cell tumor	All persons	–		–		–		4.5	−17.4 to 32.2
	Males	–		–		–		5.0	−20.1 to 38.0
	Females	–		–		–		–	
Chordoma	All persons	–		2.2	−8.3 to 14.0	−5.3	−19.8 to 11.9	−1.5	−13.0 to 11.5
	Males	–		2.6	−9.3 to 16.0	−7.0	−23.0 to 12.4	0.9	−10.2 to 13.4
	Females	–		−11.3	−27.6 to 8.7	–		−7.3	−23.9 to 12.7
Malignant ameloblastoma	All persons	–		–		–		−5.9	−23.7 to 16.2
	Males	–		–		–		–	
	Females	–		–		–		–	
Other specified sarcoma	All persons	−17.4*	−30.5 to −1.8	7.2	−8.5 to 25.5	15.5	−7.2 to 43.6	1.6	−8.2 to 12.5
	Males	−4.2	−20.6 to 15.6	−16.5	−35.7 to 8.4	9.0	−11.8 to 34.7	3.4	−5.8 to 13.5
	Females	–		2.1	−13.6 to 20.5	–		−1.9	−12.7 to 10.2
Sarcoma, NOS	All persons	–		–		–		10.1*	4.3–16.1
	Males	–		–		–		14.4*	0.5–30.1
	Females	–		–		–		6.1	−7.3 to 21.5
Other malignancy	All persons	–		–		10.1	−9.8 to 34.5	8.3	−4.1 to 22.3
	Males	–		–		−5.1	−28.1 to 25.2	4.5	−7.7 to 18.4
	Females	–		–		–		12.0	−2.6 to 28.9
All types	All persons	0.6	−3.5 to 4.8	7.4*	0.6–14.6	6.4*	2.4–10.5	4.8*	0.3–9.4
	Males	−1.0	−4.8 to 2.9	5.4	−4.2 to 16.0	5.1	−3.5 to 14.3	3.2	−2.5 to 9.1
	Females	3.0	−3.5 to 9.9	9.4*	3.6–15.5	7.4	−0.5 to 15.9	6.7*	2.5–11.2

*AAPC* average annual percent change, *CI* confidence interval, *NOS* not otherwise specified, “–” indicates calculation of the APC was precluded by at least 1 annual rate of zero

^a^The AAPC was calculated via weighted least-squares regression

* Indicates statistical significance at the 0.05 level

For all persons combined among the three age groups, only the incidence rates of Groups II and III rose significantly (AAPC 7.4 and 6.4 %, respectively). Significant increasing trend was only found in females of Group II.

For the subtypes among the three age groups, only the incidence rates of CS in Group III rose significantly (AAPC 8.2 %); in contrast, it declined significantly for other specified sarcoma in Group I (AAPC −17.4 %). Accounting for genders, the incidence rate rose most significantly for females with OS and ES in Group I (AAPC 14.0 and 14.6 %, respectively).

## Discussion

This is the first report documenting the incidence of primary BC in three age groups in Asia. The overall age-specific incidence patterns reported in this study were consistent with those reported in Western countries,^2^,^3^,^12^–^20^ showing that OS and ES are more likely to be diagnosed in youth, whereas CS and GCT occur more frequently in middle-aged and elderly individuals, and chordomas and ameloblastomas predominantly affect elderly patients. However, more details remain to be clarified.

The ASR of OS was 3.01 per million in the current study (Table 1), which is consistent with the findings in the previous large-scale studies.^3^,^12^–^14^ Further comparison of the incidences of BCs in the three age groups showed that the greatest variation in incidence was observed in the elderly and was markedly lower than the world average (2.27 and 2.39 vs. 4.6 and 3.3 per million for males and females, respectively).^5^ This finding has been reported to be associated with a lower risk of Paget’s disease (which resulted in secondary transformation into OS in the elderly) among Asians compared with Caucasian Americans.^2^,^4^,^5^ Vitamin D deficiency may also have a role involving the lower incidence in elderly Asians.^5^ As vitamin D deficiency has been associated with the risk of developing colon, prostate, breast, and several other cancers,^21^,^22^ it is theoretically possible that abundant sunshine in Taiwan along with adequate intake of vitamin D in the elderly may contribute to the reduced risk of OS.^5^,^23^

The incidence of CS reported is approximately 2–3 per million, and most literature indicates that racial differences are not significant.^3^,^12^,^13^ In contrast, other researchers have concluded that the relative frequency of CS is higher among Americans than Asians.^4^ Our observation based on the 244 cases of CS were consistent with the latter findings. Specifically, we found the ASR was only 1.20 per million and was similar to the results in China and Japan (0–1 per million),^4^,^14^ but much lower than that in the US (2.4–2.7 and 1.6–2.7 for males and females, respectively)^3^ and England (1.7–2.0 per million).^12^

Racial disparities in incidence were also evident for ES.^4^,^15^ The most striking example was from the US, which showed that there was up to a ninefold difference between black and white (ASR 0.17 vs. 1.55 per million).^15^ Similarly, the ASR of ES was only 0.52 per million in our findings, which was consistent with the results from China (0–1 per million),^14^ but 2- to 3-fold lower than the US and England.^3^,^12^

Studies reporting the incidence of malignant GCTs of bone were limited. Based on the analysis of 40 cases in the current study, the ASR was 0.20 per million in Taiwan (Table 1), thus comprising 3 % of all malignant BCs. Indeed, the incidence was slightly higher than previously reported in the US (0.16 per million).^17^ Notably, a higher incidence of GCTs in the Chinese population has been reported, ranging from 13.7 to 20 % of all bone tumors (benign and malignant).^16^,^18^ In contrast, the incidence was only 5 % in other large-scale studies.^16^–^18^ When compared with all GCTs that most frequently occur in persons aged 20–40 years,^16^ malignant GCTs in the current study were more likely to occur in the elderly, and the incidence was consistent with previous reports that showed no gender predilection.^17^ However, a limitation of the study that the data from TCR included ‘malignant’ GCTs only should be borne in mind because the distribution of malignant GCTs across age groups remains unknown.

The annual incidence of chordomas in the US is 0.80–0.84 per million.^19^,^20^ The occurrence of chordomas is rare among African Americans compared with Caucasians in the US,^19^ and has been considered to have racial disparities. Based on the analysis of 83 extracranial chordomas, the incidence patterns demonstrated herein did not reveal evidence of variations compared with Western countries.^12^–^14^,^19^,^20^ The ASR was 0.40 per million, with a pronounced male predilection.

Our study showed an increasing trend in the incidence of BC among females in Group II, and the incidences of OS and ES among females in Group I (Table 3). A strong association between growth spurts of children and adolescents and the development of OS is well known. However, this condition was not applicable for interpreting the observation for Group II. Many authors indicated that the incidence of certain types of female cancers were influenced by reproductive factors.^24^,^25^ The number of babies who were born to females aged 15–49 years (childbearing years) has been decreasing over the study period in Taiwan, with a total fertility rate of only 0.895 in 2010 (the lowest in the world).^26^ In which, first pregnancy at older age and more women receiving fertility treatment were also found.^27^ Another issue that may need more attention is the use of traditional Chinese medicine in the pediatric population for helping grow and treat musculoskeletal injury in Taiwan,^28^ because the long-term influence on health and efficacy has never been well studied. Furthermore, more Taiwanese children are raised on the incorporation of a Westernized diet and dietary factors are thought to account for approximately 30 % of cancers in Western countries.^29^,^30^ Additional investigations are warranted to clarify the association between reproductive and diet/environmental factors and risk for BC among females in Group II, and the risk for OS and ES among females in Group I.

There were limitations in this study. First, the reliability of the study may be challenged by a small number of BC subtype cases other than OS, CS, and ES, especially when they were divided into three age groups. Second, some specific diagnostic dilemmas for primary BCs existed, such as CS was notoriously hard to be diagnosed histologically, benign cartilage lesions can be difficult to differentiate from slow-growing and low-grade CS, and giant cell-rich OS may focally closely mimic malignant GCT. Third, the upward trend in the incidence among females should also be interpreted cautiously because of the number of cancers categorized into ‘other specified sarcoma’ was correspondingly decreased (AAPC −17.4 %, Group I; Table 3) as the diagnostic accuracy increased. We could not rule out the possibility that an increased level of diagnostic specificity led to a relative increase in the proportion of certain subtype in the same age group. Fourth, the completeness of cancer registration, as the DCO% fell from 2.66 % in 2003 to 0.85 % in 2010 (indicating a 1.8 % increase of the registration completeness during the 8-year study period), may have partly contributed to the increasing trend. Furthermore, the incidence data of Chinese available for comparison in the current study were from different registries in China,^14^ and with a wide variation in MV% (49.8 % [Shanghai] to 97.2 % [Zhongshan] for primary BCs). As a result, the accuracy in judgments of the incidence variations between countries has been limited.

## Conclusion

The estimates of primary BC incidence rates among three age groups presented herein were based, for the first time, on high-quality national registration data in Taiwan. Our study demonstrates distinct differences in distribution of BC subtypes and incidence among three age groups. We have confirmed some historic findings from other series that indicated significant variations in primary BC incidence according to age, gender, and race. The results of this study may lay the groundwork for generating further research hypotheses into causes of these cancers.
